# *Thymelaea hirsuta* (L.) Endl*.* extract attenuates NLRP3 inflammasome activation via modulation of ATPase activity

**DOI:** 10.3389/fphar.2026.1781860

**Published:** 2026-04-15

**Authors:** Seongjong Lee, Seoyeon Jang, Hangyeol Lee, Ali Khorchani, Abdelhamid Khaldi, Mi Jin Lee, Dong Keun Yi, Soo-Yong Kim, Sangho Choi, Hyungseok Seo, Man S. Kim, Jin Hyub Paik, Hyung Won Ryu, Yoonsung Lee, Yong Hwan Park

**Affiliations:** 1 BK21 R&E Initiative for Advanced Precision Medicine, Ajou University School of Medicine, Suwon, Republic of Korea; 2 Department of Microbiology, Ajou University School of Medicine, Suwon, Republic of Korea; 3 Department of Biomedical Sciences, Graduate School of Ajou University, Suwon, Republic of Korea; 4 Department of Biomedical Science and Technology, Kyung Hee University, Seoul, Republic of Korea; 5 School of Medicine, Clinical Research Institute, Kyung Hee University Hospital at Gangdong, Kyung Hee University, Seoul, Republic of Korea; 6 Natural Product Research Center and Natural Product Central Bank, KRIBB, Cheongju-si, Chungcheongbuk-do, Republic of Korea; 7 Laboratory of Management and Valorization of Forest Resources, The National Research Institute of Rural Engineering, Water, and Forestry. INRGREF. University of Carthage, Ariana, Tunisia; 8 International Biological Material Research Center, Korea Research Institute of Bioscience and Biotechnology, Daejeon, Republic of Korea; 9 Department of Anesthesiology and Pain Medicine, Kyung Hee University Hospital at Gangdong, College of Medicine, Kyung Hee University, Seoul, Republic of Korea; 10 Center for Space Biomedical Sciences, NEXUS Institute, Kyung Hee University, Seoul, Republic of Korea

**Keywords:** apoptosis-associated speck-like protein containing a CARD, caspase-1, interleukin-1β, NLRP3 inflammasome, Thymelaea hirsuta (L.) Endl

## Abstract

**Background:**

The NLRP3 inflammasome is a multiprotein complex of the innate immune system that mediates the maturation and secretion of interleukin-1β (IL-1β) and plays a pivotal role in the pathogenesis of chronic inflammatory diseases, including asthma, metabolic disorders, and autoimmune diseases. However, natural product–derived metabolites that directly modulate NLRP3 activation remain limited. This study aimed to investigate the inhibitory effects of *Thymelaea hirsuta* extract (TMH) on NLRP3 inflammasome activation and to elucidate its underlying mechanism of action.

**Methods:**

TMH was prepared by methanol extraction and chemically profiled using UPLC-QTOF/MS analysis. Human monocytic THP-1 cells and murine macrophage J774A.1 cells were used to evaluate inflammasome activation. Following lipopolysaccharide (LPS) priming, cells were stimulated with ATP or nigericin to induce NLRP3 inflammasome activation. IL-1β secretion was quantified by ELISA, and the expression of NLRP3 inflammasome-related proteins was evaluated by Western blot analysis. Cell viability was assessed using the EZ-CYTOX assay.

**Results:**

TMH exhibited no cytotoxicity at concentrations up to 100 μg/mL. TMH treatment (10, 50, and 100 μg/mL) significantly reduced LPS/ATP- or LPS/nigericin-induced IL-1β secretion. However, even at the highest concentration tested (100 μg/mL), TMH did not significantly affect pro-IL-1β expression or NF-κB signaling, indicating that the priming step was not altered. Instead, TMH suppressed NLRP3 inflammasome activation during the activation phase. In addition, TMH treatment at 1 μg/mL was associated with reduced NLRP3 ATPase activity, suggesting a potential effect on inflammasome assembly.

**Conclusion:**

TMH attenuated NLRP3 inflammasome activation without affecting the priming step. These findings suggest that TMH may act as a natural product–derived modulator of NLRP3-mediated inflammatory responses.

## 1 Introduction

Chronic inflammatory diseases are increasingly associated with dysregulated activation of the NLRP3 inflammasome. This activation occurs in two distinct steps: The priming signal (signal 1) is typically induced by pathogen-associated molecular patterns such as LPS, which activate NF-κB signaling and upregulate NLRP3 and pro- IL-1β expression (Kelley et al., 2019). The second signal is triggered by stimuli such as extracellular ATP, microbial toxins, or crystalline particles, which induce cellular stress responses including potassium efflux (Muñoz-Planillo et al., 2013) and reactive oxygen species (ROS) (Zhou et al., 2011) and inflammasome assembly. These events promote the assembly of the inflammasome complex composed of NLRP3, apoptosis-associated speck-like protein containing a CARD (ASC), and pro-caspase-1 (Fu and Wu, 2023), leading to the maturation and secretion of pro-inflammatory cytokines, including IL-1β and IL-18. Dysregulation of this pathway has been implicated in various pathologies, including arthritis (Guo et al., 2018), diabetes (Ding et al., 2019), Alzheimer’s disease (Barczuk et al., 2022; Liang et al., 2022), neutrophilic asthma (Simpson et al., 2014; Quoc et al., 2024) and cryopyrin-associated periodic syndromes (Booshehri and Hoffman, 2019).

Natural products are increasingly recognized as valuable resources for the development of NLRP3 inflammasome inhibitors because of their structural diversity and generally favorable safety profiles compared to synthetic alternatives. Numerous plant-derived secondary metabolites have been reported to exert anti-inflammatory effects by modulating innate immune signaling pathways, suggesting that botanical extracts may represent promising sources of novel modulators of inflammasome activation.

*Thymelaea hirsuta* (L.) Endl., commonly known as the tangled sparrow plant, is an evergreen shrub of the Thymelaeaceae family. Native to the arid coastal regions of the Mediterranean and North Africa, it has long been used in ethnomedicine to treat diverse ailments such as dermatophytosis, hypertension, and diabetes. The therapeutic potential of *T*. *hirsuta* is largely attributed to its rich phytochemical composition, including flavonoids, saponins, coumarins, and tannins (Elhady et al., 2021; Nocera et al., 2021), which contribute to its anti-aging, neuroprotective, and skin-healing properties. In addition, *T*. *hirsuta* has been traditionally used for anti-inflammatory properties (Boudjelal et al., 2013; Azza and Oudghiri, 2015; Merghem et al., 2020). Recent pharmacological studies have validated several traditional therapeutic uses of this plant. The aqueous extract and ethyl acetate fraction of *T*. *hirsuta* demonstrated significant anti-diabetic and pancreas-protective effects by inhibiting intestinal α-glucosidase activity and promoting insulin secretion (Abid et al., 2021). Furthermore, the plant exhibited hepatoprotective activity and normalized serum aminotransferase levels in carbon tetrachloride-induced liver injury models (Azza et al., 2012). In the context of cancer therapy (Toumi et al., 2023), the ethyl acetate fraction has shown promise against colorectal cancer by inducing cell cycle arrest and apoptosis through the downregulation of integrin α5 expression and focal adhesion kinase phosphorylation. Recent findings suggest potential anti-obesity effects (Abdallah et al., 2023) via lipase inhibition, highlighting the metabolic regulatory properties of the plant. Although *T*. *hirsuta* has been reported to exhibit antimicrobial, antioxidant, and general anti-inflammatory activities, its specific effects on NLRP3 inflammasome–mediated inflammatory responses have not yet been investigated.

Although *T*. *hirsuta* is known to possess bioactive properties, its specific effect on the NLRP3 inflammasome has not been defined. Therefore, this study investigated whether TMH inhibits NLRP3 inflammasome activation and explored its potential relevance in NLRP3-driven inflammatory conditions.

## 2 Materials and methods

### 2.1 Plant sample collection

The plant material was collected in April 2019 from the Kairouan region of Tunisia and was taxonomically identified based on morphological characteristics by Dr. Ali Khorchani voucher specimen (KRIB 0085722) has been deposited in the herbarium of the Korea Research Institute of Bioscience and Biotechnology (KRIBB, Daejeon, Republic of Korea). All plant materials used in this study were collected and utilized in accordance with institutional, national, and international guidelines and regulations in Tunisia. All necessary permits and licenses were obtained to comply with ethical and legal requirements.

After shade-drying, the dried leaves were mechanically ground, and 100 g of the powdered material was extracted with 1 L of methanol (99.9%, HPLC grade) using an ultrasonic extractor (SDN-900H, SD-ULTRASONIC CO., LTD). The extraction was performed at 40 kHz and 1500 W, and the procedure was repeated 30 times using freshly added methanol for each cycle. Each extraction cycle consisted of 15 min of ultrasonication followed by a 120 min standing period at room temperature. The combined extracts were filtered using filter paper (Qualitative Filter No. 100, HYUNDAI MICRO CO., LTD) and subsequently concentrated under reduced pressure at 40 °C using a rotary evaporator until dryness. A total of 2 g of dried methanolic extract was obtained. Based on the weight of the initial dried plant material (100 g) and the final dried extract (2 g), the native drug–extract ratio (DER native) was calculated to be 50:1 (w/w).

The methanolic extract of TMH (FBM344-100) used in this study was provided by the International Biological Material Research Center of KRIBB, which maintains documentation on the legal origin of biological material. The extract was originally prepared from dried leaves of TMH by the contributing authors and subsequently deposited at the center, from which it was supplied for the present study. No additional collection permits were required for the present study. The dried extract was dissolved in dimethyl sulfoxide (DMSO) to prepare stock solutions for subsequent biological experiments.

### 2.2 UPLC-QTOF/MS conditions

UPLC-QTOF/MS data were acquired on a Xevo G2-S QTOF mass spectrometer (Waters) coupled to an ACQUITY UPLC I-Class system equipped with a PDA detector. Separation was performed on an ACQUITY UPLC BEH C18 column (100 × 2.1 mm, 1.7 μm) at 35 °C with a flow rate of 0.4 mL/min and an injection volume of 2 μL. The mobile phases were (A) water with 0.1% formic acid (v/v) and (B) acetonitrile with 0.1% formic acid (v/v). The gradient was: 5% B for 0–1 min, 5%–100% B from 1 to 22.3 min, return to 5% B at 22.4 min, and re-equilibration at 5% B until 25 min. Mass spectrometry was performed using ESI with a source temperature of 110 °C and desolvation temperature of 350 °C. The capillary and cone voltages were 2.3 kV and 40 V, respectively, with desolvation and cone gas flows of 800 and 50 L/h. Leucine–enkephalin (*m/z* 556.2771, ESI+) was used as the lock-mass for real-time mass correction. Data were collected in DDA mode over m/z 100–1500 and processed using MassLynx software.

### 2.3 Reagents and materials

Cell culture reagents, including Dulbecco’s Modified Eagle Medium (DMEM), Roswell Park Memorial Institute 1640 (RPMI 1640), fetal bovine serum (FBS), and penicillin–streptomycin, were obtained from GenDEPOT (United States). Phorbol 12-myristate 13-acetate (PMA; HY-18739) and imiquimod (IMQ; HY-B0180) were purchased from MedChem Express (United States). N-acetyl-L-cysteine (NAC; A9165) and disuccinimidyl suberate (DSS; S1885) were obtained from Sigma-Aldrich (United States). The EZ-CYTOX cell viability assay kit (EZ-500) was purchased from DoGenBio (Republic of Korea).

For inflammasome stimulation, ultrapure LPS (tlrl-ppglps), nigericin (tlrl-nig), adenosine triphosphate (ATP; tlrl-atpl), and flagellin (FLA-ST; tlrl-epstfla) were obtained from InvivoGen (United States).

Primary antibodies against α-tubulin (sc-5286) and ASC (sc-514414) were purchased from Santa Cruz Biotechnology (United States). NF-κB p65 (8242) antibody was obtained from Cell Signaling Technology (United States). Antibodies specific for human IL-1β (AF-401-NA) and mouse IL-1β (AF-201-NA) were purchased from R&D Systems (United States). Highly cross-adsorbed secondary antibody against mouse IgG (H + L) (A32723) was obtained from Invitrogen (United States). Additional reagents used included Lipofectamine™ 2000 (11668019; Invitrogen, United States), a phosphatase inhibitor cocktail (P3200-005; GenDEPOT, United States), and a protease inhibitor cocktail (PPI1015; Quartett GmbH, Germany). Mitochondrial ROS were detected using the MitoSOX™ Red mitochondrial superoxide indicator (M36005; Invitrogen, United States). Nuclei were counterstained with mounting medium containing DAPI (ab104139; Abcam, United Kingdom). For reporter assays, the Bright-Glo™ Luciferase Assay System (E2610), pGL4.32 [luc2P/NF-κB-RE/Hygro] vector (E8491), and the ADP-Glo™ Max Assay (V7001) were purchased from Promega (United States).

### 2.4 Cell culture and stimulation

THP-1 and J774A.1 cells were obtained from the Korean Cell Line Bank and used between passages 5 and 20. All cells were cultured under standard conditions following ATCC guidelines. For differentiation, THP-1 monocytes were differentiated into macrophage-like cells by treatment with PMA (500 nM) for 3 h. Following differentiation, cells were washed three times with PBS to remove residual PMA and subsequently incubated in fresh complete medium for 48 h as a resting period prior to experimental treatments (Park et al., 2020). J774A.1 cells were seeded overnight before experiments. For inflammasome priming, cells were stimulated with LPS (100 ng/mL) for 5 h. TMH (10, 50, or 100 μg/mL) was added 3 h after LPS stimulation and incubated for an additional 2 h before the activation step (Lee et al., 2024a; Lee et al., 2024b). TMH was dissolved in DMSO to prepare a stock solution and subsequently diluted in culture medium to obtain the desired treatment concentrations. The final concentration of DMSO in all experiments was maintained at ≤0.1% (v/v). Cells treated with an equivalent concentration of DMSO were used as the vehicle control.

NLRP3 inflammasome activation was subsequently induced by stimulation with nigericin (10 μM) or ATP (5 mM) for 30–60 min, or with imiquimod (200 μM) for 1 h, as indicated. To activate the AIM2 or NLRC4 inflammasomes, cells were transfected with double-stranded DNA (1 μg/mL) or flagellin (1 μg/mL), respectively, using Lipofectamine™ 2000 (Invitrogen, United States) for 3 h. IL-1β secretion in culture supernatants and intracellular pro-IL-1β were analyzed by immunoblotting.

### 2.5 ELISA

IL-1β levels in culture supernatants were quantified using a commercially available ELISA kit (mouse IL-1β: DY401, human IL-1β: DY201, R&D system, United States) according to the manufacturer’s instructions. Briefly, cell culture supernatants were collected and centrifuged to remove residual cells and debris. Samples were added to antibody-coated plates, followed by incubation with a detection antibody and substrate solution. Absorbance was measured at 450 nm, and cytokine concentrations were calculated using a standard curve.

### 2.6 Cell viability assay

Differentiated THP-1 cells (0.45 × 10^5^ cells/well) were seeded in 96-well plates 2 days prior to the assay, and J774A.1 cells (0.4 × 10^5^ cells/well) were seeded 1 day prior to the assay. Cells were treated with TMH (10, 50, or 100 μg/mL) for 2 h in J774A.1 cells or for 18 h in differentiated THP-1 cells. Cell viability was assessed using EZ-CYTOX reagent (DoGenBio, Republic of Korea), which was added and incubated for 30 min (J774A.1) or 1 h (THP-1). Absorbance was measured at 450 nm using an iMark™ Microplate Reader (Bio-Rad, United States). Different treatment durations were used to account for the distinct proliferation characteristics of each cell line: J774A.1 macrophages remain proliferative during incubation, whereas PMA-differentiated THP-1 macrophages behave as post-mitotic cells.

### 2.7 NF-κB reporter gene assay

HEK293FT cells (2 × 10^4^ cells/well) were seeded in white 96-well plates 1 day prior to transfection. Cells were transfected with the NF-κB responsive pGL4.32 luciferase reporter vector (0.1 μg/well) using Lipofectamine™ 2000. After 24 h, cells were stimulated with tumor necrosis factor alpha (TNF-α) (20 ng/mL) for 5 h to induce NF-κB activation, in the presence or absence of TMH (10, 50, or 100 μg/mL). Luciferase activity was measured using the Bright-Glo™ Luciferase Assay System (Promega, United States) and quantified with a FLUOstar OPTIMA microplate reader (BMG Labtech, Germany). To minimize experimental variation in transfection efficiency, cells were seeded at a 2 × 10^4^ cells/ 96 well plate and transfection mixtures were prepared as a master mix. Luciferase activity was normalized to total protein concentration to account for differences in cell number and transfection efficiency. Experiments were performed in triplicate and repeated three times independently. Data are presented as relative fold change compared to the untreated control group.

### 2.8 Cytoplasmic and nuclear protein fractionation

J774A.1 cells (8 × 10^6^ cells/plate) were primed with LPS and treated with TMH (50 or 100 μg/mL) for 2 h. Cells were harvested, washed with PBS, and resuspended in ice-cold CER I buffer. After incubation with CER II and centrifugation, the supernatant containing the cytoplasmic fraction was collected. The remaining pellet was subsequently resuspended in ice-cold NER buffer to extract nuclear proteins. Cytoplasmic and nuclear fractions were subsequently subjected to SDS-PAGE followed by immunoblotting.

### 2.9 Intracellular ROS measurement

J774A.1 cells (2 × 10^5^ cells/well) were seeded in 4-well culture slides 1 day prior to the experiment. Cells were primed with LPS (100 ng/mL) for 5 h and then treated with TMH (50 μg/mL) for 2 h. After washing with DPBS, cells were incubated with MitoSOX™ Red (1 μM) for 10 min at 37 °C in the dark. Cells were washed again, stimulated with ATP (5 mM) for 5 min, fixed with 4% paraformaldehyde (PFA), and mounted using medium containing DAPI. Images were captured using a confocal laser scanning microscope (LSM710, Carl Zeiss, Germany).

### 2.10 ASC oligomerization and speck immunofluorescence

THP-1 cells (1.2 × 10^6^ cells/well) were washed with PBS and treated with disuccinimidyl suberate (DSS, 2.5 mM) for 30 min at 25 °C to induce ASC oligomerization, which was then analyzed via immunoblotting. ASC speck formation was visualized in J774A.1 cells (2 × 10^5^ cells/well) following NLRP3 inflammasome activation. Cells were fixed with 4% PFA, permeabilized using Triton X-100, and blocked with BSA and glycine. ASC staining was performed using an anti-ASC antibody followed by AF488-conjugated anti-mouse IgG secondary antibody. Nuclei were counterstained with DAPI-containing mounting medium. Confocal microscopy was used to acquire images.

### 2.11 NLRP3 ATPase activity assay

Human recombinant NLRP3 (0.139 mg/mL, BPS Bioscience, United States) was incubated with TMH or vehicle control (DMSO) in the reaction buffer at RT for 3 h. Subsequently, ATP (500 μM, ultra-pure ATP) was added, and the mixture was incubated for additional 40 min at 37 °C. ATP hydrolysis by NLRP3 was quantified using ADP-Glo Max assay (Promega, United States) according to the manufacturer’s protocol.

### 2.12 Zebrafish husbandry and TMH preparations

All zebrafish experiments were approved by the Institutional Animal Care and Use Committee (IACUC) of Kyung Hee University Hospital at Gangdong (IACUC approval no. KHNMC AP 2024-009) and conducted in accordance with institutional guidelines. Wild-type and Tg (*mpeg1:*EGFP) (Ellett et al., 2011) zebrafish embryos were maintained at 28.5 °C under standard laboratory conditions. For preparation of the natural product, the powdered extract was dissolved in 100% DMSO to a final concentration of 40 mg/mL by vortexing thoroughly. The stock solution was aliquoted and stored at −20 °C.

### 2.13 Embryo survival assay

Zebrafish embryos at 24 h post-fertilization (hpf) were dechorionated prior to pigmentation using pronase. After dechorionation, 30 embryos were transferred to 60-mm Petri dishes containing 6 mL of E3 medium and treated with TMH at concentrations of 1, 10, or 100 μg/mL. Embryos were washed twice with fresh E3 medium prior to treatment. For vehicle controls, DMSO was added to a final concentration of 0.25% (v/v), corresponding to the DMSO content of the highest TMH concentration. Embryo survival was assessed daily from 1 to 5 days post-fertilization (dpf), and treatment solutions were replaced with fresh solutions every 24 h for a total of 5 days.

### 2.14 Inflammation induction and TMH treatment in zebrafish

Inflammation was induced using an LPS immersion method. LPS (L2880, Sigma-Aldrich) was dissolved in ultrapure water by vortexing to prepare a stock solution and diluted a final concentration of 10 μg/mL in E3 medium. TMH stock solution (40 mg/mL in 100% DMSO) was diluted in E3 medium containing 1-phenyl-2-thiourea (PTU) to a final concentration of 1 μg/mL. Control solutions contained DMSO at a final concentration of 0.0025% (v/v). Twenty embryos at 24 hpf were transferred to a 60-mm Petri dish containing 6 mL of treatment solution. Embryos were incubated under treatment conditions, with fresh solutions replaced every 24 h. At 72 hpf, embryos were fixed with 4% PFA overnight at 4 °C.

### 2.15 Sudan black B staining

Embryos fixed overnight in 4% PFA at 4 °C were washed three times with 1x phosphate buffered saline (PBS). Samples were then incubated with Sudan black B working solution (prepared by mixing Sudan black B stock, phenol, and 70% ethanol) for 1 h at RT with gentle rocking, protected from light. Next, embryos were washed with 70% ethanol and subsequently transferred to PBS-T containing depigment solution (30% KOH and 3% H_2_O_2_) for 5 min. Samples were rinsed twice with PBS-T and stored in PBS at 4 °C, and imaged using a Leica M205 C microscope.

### 2.16 Whole-mount *in situ* hybridization

Digoxigenin (DIG)-labeled antisense RNA probes were synthesized using the DIG-RNA Labeling Kit (SP6/T7; Roche) and diluted 1:50 in hybridization buffer (Formamide, torula RNA powder, 20 X SSC, 1 M Citric acid, 20% Tween-20 and Heparin). Embryos fixed overnight in 4% PFA were dehydrated and stored at −20 °C. Following rehydration and PBS-T washes, embryos were subjected to permeabilization and pre-hybridization steps before incubation with RNA probes. After hybridization, embryos were washed with 2X SSC and 0.2X SSC buffer and incubated with anti-DIG-AP-conjugated Fab fragments (1:5000) at 4 °C overnight. After the antibody removed and washing with PBS-T and Alkaline Phosphatase (AP) buffer, color development was performed using AP solution containing NBT/BCIP and stopped with a solution containing 1 mM EDTA (pH 5.2). Finally, embryos were stored in methanol at 4 °C, protected from light. Images were acquired using a Leica M205 C microscope.

## 3 Results

### 3.1 TMH inhibits NLRP3 inflammasome activation without cytotoxicity

**FIGURE 1:**
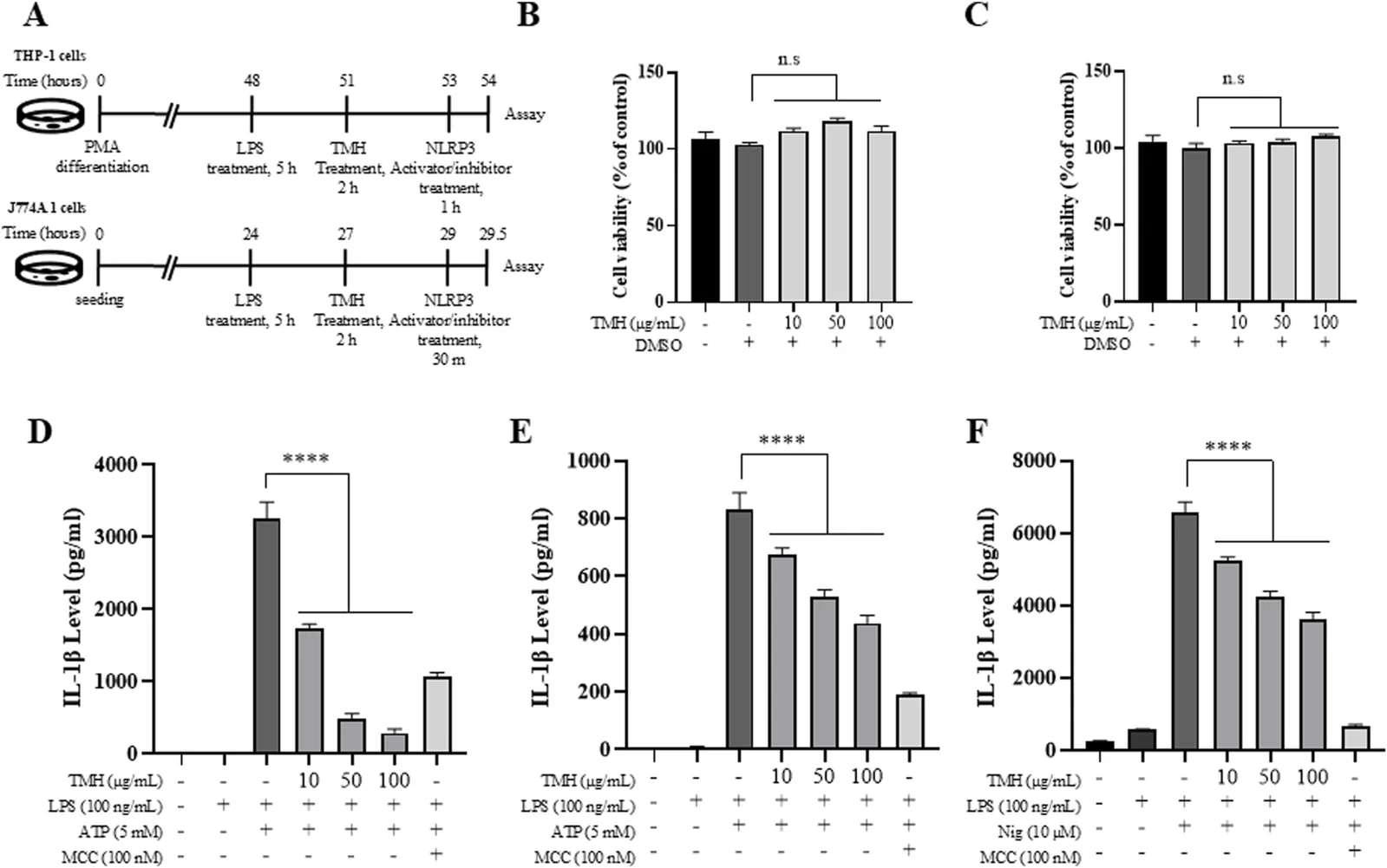
TMH inhibits activation of the NLRP3 inflammasome. **(A)** Schematic workflow of *in vitro* NLRP3 inflammasome activation. **(B,C)** Cell viability was assessed in J774A.1 cells **(B)** and PMA (500 nM)-differentiated THP-1 cells **(C)** treated with TMH for 2 or 18 h using the EZ-Cytox assay. **(D)** LPS-primed J774A.1 cells were treated with TMH for 2 h, then stimulated with ATP (5 mM) for 30 min in the presence or absence of MCC950. IL-1β (p17) levels in supernatants were measured by ELISA. **(E,F)** PMA-differentiated THP-1 cells were primed with LPS, treated with TMH for 2 h, then stimulated with ATP (5 mM) **(E)** or nigericin (10 μM) **(F)** for 1 h, with or without MCC950. IL-1β (p17) levels in supernatants were determined by ELISA. Data are presented as the mean ± SD (n = 3). Statistical analysis was performed using one-way ANOVA followed by Tukey’s multiple comparisons test. *P < 0.05, **P < 0.01, ***P < 0.001, ****P < 0.0001; n.s., not significant.

Figure 1A illustrates the experimental timeline for TMH treatment and NLRP3 inflammasome activation in macrophages. To exclude nonspecific effects due to cytotoxicity, the impact of TMH on cell viability was first examined. TMH treatment at concentrations up to 100 μg/mL did not affect the viability of either J774A.1 or PMA-differentiated THP-1 cells (Figures 1B,C).

To examine the effect of TMH on NLRP3 inflammasome activation, LPS-primed J774A.1 cells were treated with ATP, a canonical NLRP3 activator. TMH treatment (10, 50, 100 μg/mL) induced a significant dose-dependent reduction in IL-1β secretion (n = 3, one-way ANOVA followed by Tukey’s multiple comparisons test, p < 0.01) (Figure 1D). The calculated IC_50_ value for inhibition of IL-1β secretion was approximately 9.4 μg/mL, indicating potent suppression of NLRP3 inflammasome activation by TMH. At higher concentrations, the inhibitory effect was comparable to that of MCC950 (Tapia-Abellán et al., 2019; Li et al., 2022), a well-established NLRP3 inhibitor. Immunoblot analysis demonstrated that TMH did not alter the protein levels of pro-IL-1β or α-tubulin, indicating that it did not interfere with the priming step of inflammasome activation (Supplementary Figure 1A).

These findings were reproduced in PMA-differentiated THP-1 cells stimulated with ATP (n = 3, one-way ANOVA followed by Tukey’s multiple comparisons test, p < 0.01) (Figure 1E; Supplementary Figure 1B). In addition, TMH dose-dependently suppressed nigericin-induced IL-1β secretion (n = 3, one-way ANOVA followed by Tukey’s multiple comparisons test, p < 0.01), which activates the NLRP3 inflammasome via potassium efflux, without affecting pro-IL-1β expression (Figure 1F; Supplementary Figure 1C). Collectively, these results demonstrate that TMH suppresses NLRP3 inflammasome activation in macrophages without inducing cytotoxicity or impairing the priming phase.

### 3.2 TMH preferentially suppresses NLRP3 inflammasome activation independently of NF-κB signaling

**FIGURE 2:**
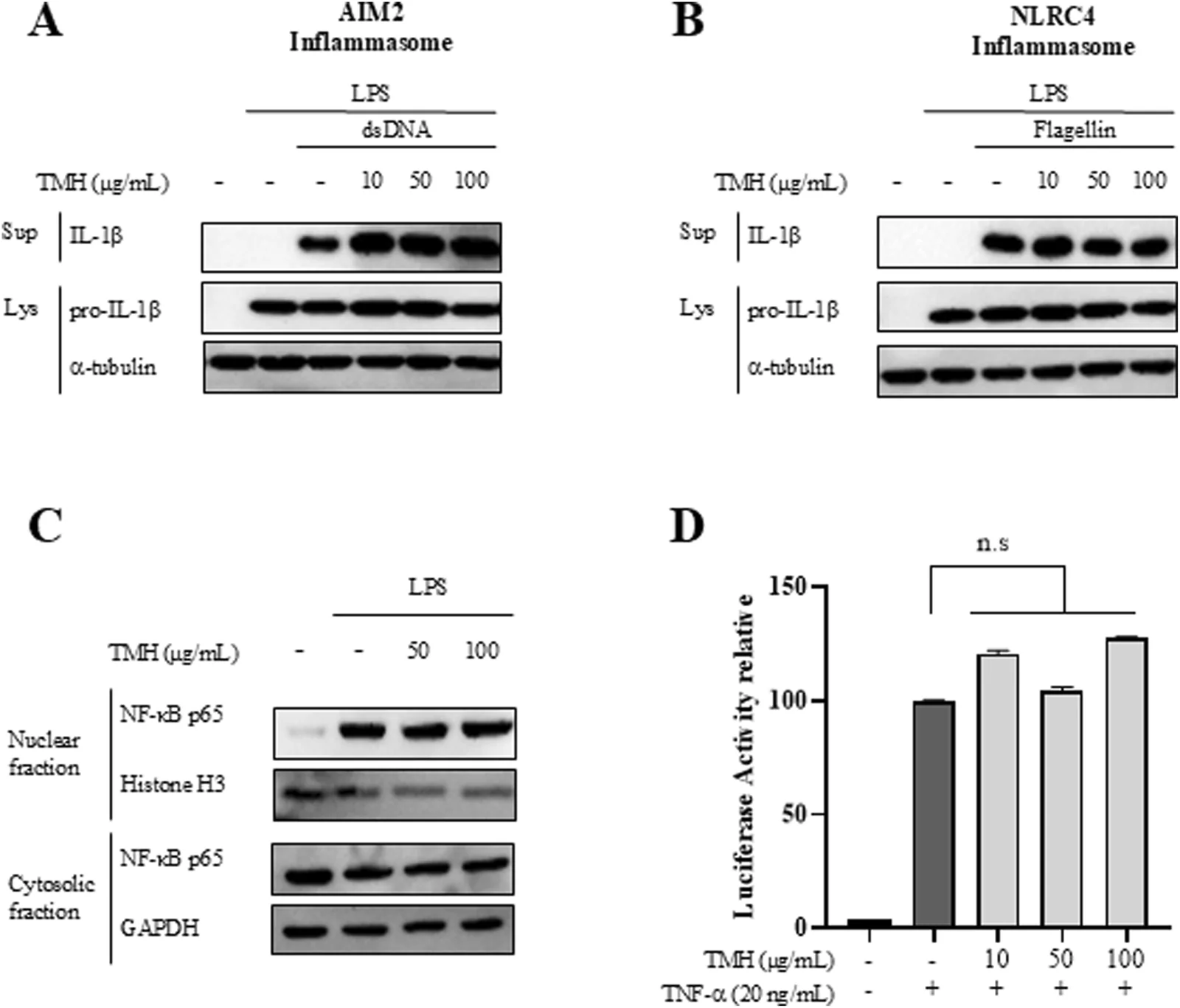
TMH does not affect other inflammasome or inflammatory pathways. **(A,B)** LPS-primed J774A.1 cells were treated with TMH for 2 h, followed by stimulation with either dsDNA (1 μg/mL) **(A)** or flagellin **(B)** transfected using Lipofectamine 2000 for 3 h. **(C)** Following TMH treatment (2 h), nuclear and cytosolic fractions of LPS-primed J774A.1 cells were separated and assessed for NF-κB p65 translocation by immunoblotting. **(D)** HEK293FT cells transfected with the pGL4.32 luciferase reporter vector were treated with TNF-α (20 ng/mL) for 5 h, with or without TMH pre-treatment (2 h). Luciferase activity was measured using the Bright-Glo™ luciferase assay system (Promega). Data are presented as the mean ± SD (n = 3). Statistical analysis was performed using one-way ANOVA followed by Tukey’s multiple comparisons test n.s., not significant.

To assess the specificity of TMH for the NLRP3 inflammasome, its effects on other inflammasome pathways were evaluated. J774A.1 cells were stimulated with dsDNA or flagellin to activate the AIM2 (Hornung et al., 2009; Sagulenko et al., 2013) and NLRC4 (Zhao et al., 2011; Matusiak et al., 2015) inflammasomes, respectively. TMH treatment did not reduce IL-1β secretion induced by either stimulus even at the highest concentration tested (100 μg/mL) (Figures 2A,B), suggesting that TMH does not broadly inhibit inflammasome activation but selectively targets the NLRP3 pathway.

As NF-κB signaling is essential for the priming step of inflammasome activation (Kaszycki and Kim, 2025), we investigated whether TMH modulates this pathway. Even at the highest concentration tested (100 μg/mL), Immunoblot analysis of cytosolic and nuclear fractions revealed that TMH did not alter LPS-induced nuclear translocation of NF-κB p65 subunit (Giridharan and Srinivasan, 2018) in J774A.1 cells (Figure 2C). Consistently, an NF-κB luciferase reporter assay performed in HEK293FT cells showed a similar level in NF-κB-dependent transcriptional activity following TMH treatment (Figure 2D). These findings indicate that TMH suppresses NLRP3 inflammasome activation without interfering with NF-κB-mediated priming.

### 3.3 TMH inhibits ASC oligomerization and attenuates NLRP3 ATPase activity

**FIGURE 3:**
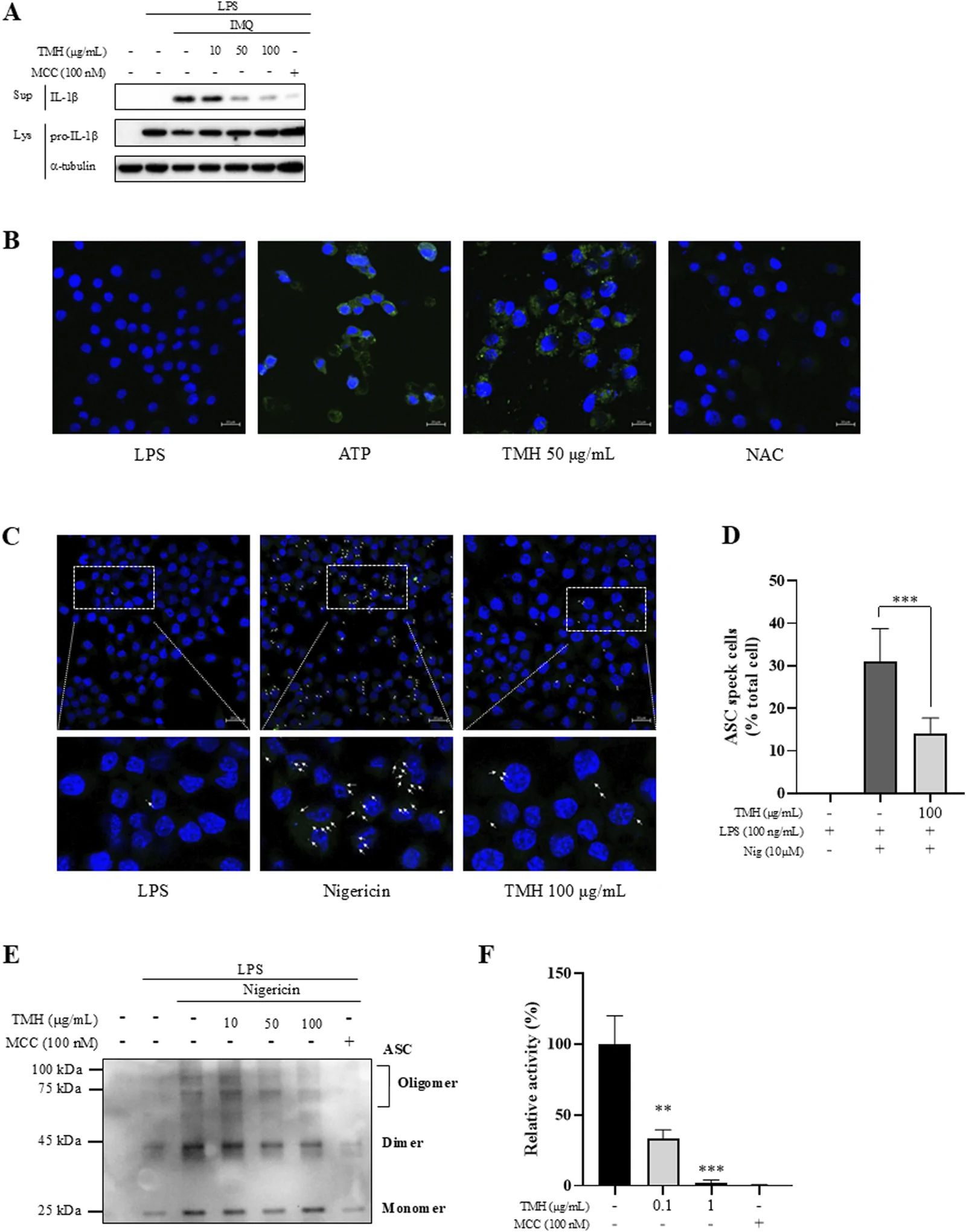
TMH inhibits NLRP3 inflammasome activation by suppressing ASC oligomerization and ATPase activity. **(A)** LPS-primed J774A.1 cells were treated with TMH for 2 h, then stimulated with imiquimod (200 μM) for 1 h. **(B)** Cells were treated with TMH (2 h), stained with MitoSOX (5 μM, 10 min), and stimulated with ATP (5 mM, 5 min). Intracellular ROS were visualized by confocal microscopy. **(C,D)** PMA-differentiated THP-1 cells were primed with LPS, treated with TMH for 2 h, then stimulated with nigericin (10 μM, 1 h). ASC speck formation was examined by confocal microscopy **(C)**, and representative images from five fields are shown **(D)**. **(E)** Cells were stimulated with or without MCC950 and cross-linked with DSS (2.5 mM, 30 min) before immunoblotting for ASC oligomerization. **(F)** NLRP3 ATPase activity was measured using the ADP-Glo™ assay to quantify ATP-to-ADP conversion following TMH treatment. Data are presented as the mean ± SD (n = 3). Statistical analysis was performed using one-way ANOVA followed by Tukey’s multiple comparisons test. **P < 0.01, ***P < 0.001.

To further elucidate the mechanism by which TMH suppresses NLRP3 inflammasome activation, we examined its effect on imiquimod-induced activation via a potassium efflux-independent pathway (Groß et al., 2016). TMH treatment (10, 50, 100 μg/mL) significantly reduced IL-1β release in response to imiquimod stimulation (Figure 3A), suggesting that its inhibitory effect is not limited to the modulation of potassium efflux.

Subsequently, we assessed whether TMH influenced intracellular ROS generation, an upstream signal associated with NLRP3 activation (Zhang et al., 2015). Confocal microscopy revealed that TMH treatment did not noticeably alter ATP-induced ROS accumulation (Cruz et al., 2007) compared with control cells (Figure 3B), indicating that ROS scavenging is unlikely to be the primary mechanism underlying inflammasome inhibition.

As inflammasome assembly requires ASC oligomerization (Lu and Wu, 2015), we examined the effects of TMH on this process. Confocal microscopy revealed that TMH reduced ASC speck formation (Hoss et al., 2017) in LPS-primed nigericin-stimulated THP-1 cells (Figures 3C,D). Chemical cross-linking assays consistently demonstrated that TMH treatment (10, 50, 100 μg/mL) inhibited ASC oligomerization in a dose-dependent manner (Figure 3E).

As the ATPase activity of the NLRP3 NACHT domain (Brinkschulte et al., 2022) is essential for inflammasome assembly, we evaluated the effect of TMH on NLRP3 ATPase activity (Lee et al., 2024a). TMH reduced ATPase activity in a concentration-dependent manner, with inhibition observed even at 1 μg/mL (Figure 3F). These results indicate that TMH suppresses NLRP3 inflammasome activation by attenuating NLRP3 ATPase activity, thereby impairing ASC oligomerization and subsequent assembly of the inflammasome complex.

### 3.4 Toxicity assessment of TMH in zebrafish embryos

**FIGURE 4:**
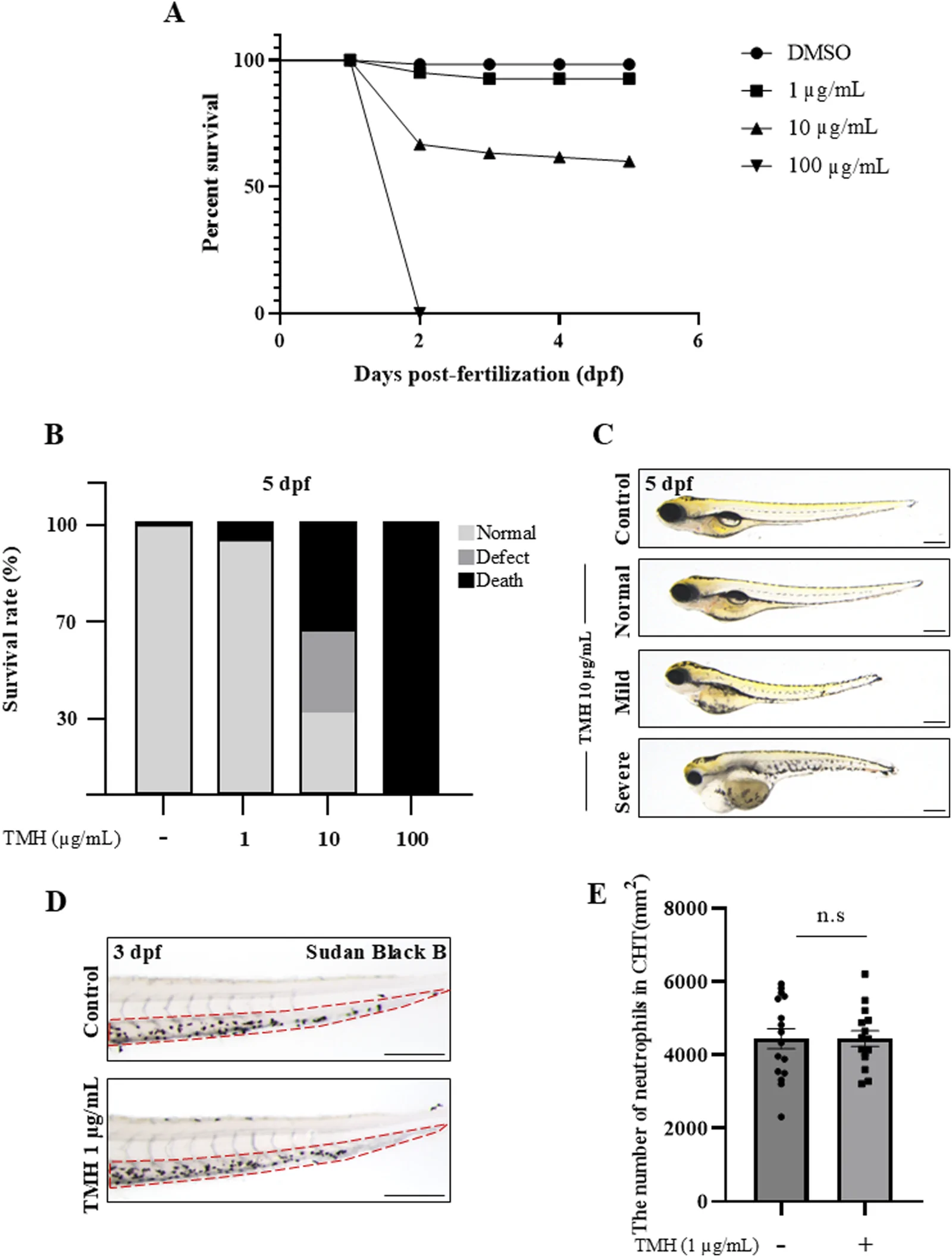
Toxicity assessment of TMH in zebrafish embryos. **(A)** Survival curves of zebrafish embryos treated with TMH at concentrations of 1, 10, or 100 μg/mL. Sixty embryos were used per condition and maintained in E3 medium. The control group was treated with DMSO (0.25%, v/v), corresponding to the highest TMH concentration. **(B)** Survival and phenotype distribution of 5 dpf embryos treated with TMH at distinct concentrations from 1 to 5 dpf. **(C)** Representative images showing developmental abnormalities observed in embryos treated with 10 μg/mL TMH at 5 dpf. **(D)** Representative images of Sudan Black B staining in 3 dpf embryos treated with vehicle control (0.0025% DMSO with PTU; upper panel) or TMH (1 μg/mL; lower panel). **(E)** Quantification of neutrophil counts in the CHT (red dashed outline) following treatment with TMH (1 μg/mL). Data are presented as mean ± SEM of individual embryos. Statistical significance was determined using an unpaired two-tailed Student’s t-test; n.s., not significant (p = 0.9997). Scale bars, 300 µm.

To evaluate the *in vivo* safety of TMH, zebrafish embryos were exposed to increasing concentrations of TMH and monitored for survival and developmental abnormalities. Treatment with 1 μg/mL TMH did not affect survival or gross morphology compared with the dimethyl sulfoxide control, whereas higher concentrations (10 and 100 μg/mL) increased mortality and developmental defects, including pericardial edema and reduced body length (Figures 4A–C).

Based on these results, 1 μg/mL was selected as the maximum non-toxic concentration for subsequent *in vivo* experiments. Neutrophil abundance was assessed using Sudan Black B staining (Le Guyader et al., 2008). No significant difference in neutrophil numbers within the caudal hematopoietic tissue (CHT) was observed between the control and TMH-treated embryos (Figures 4D,E), indicating that TMH was neither toxic nor intrinsically immunogenic at this concentration.

### 3.5 TMH suppresses LPS-induced inflammation in zebrafish

**FIGURE 5:**
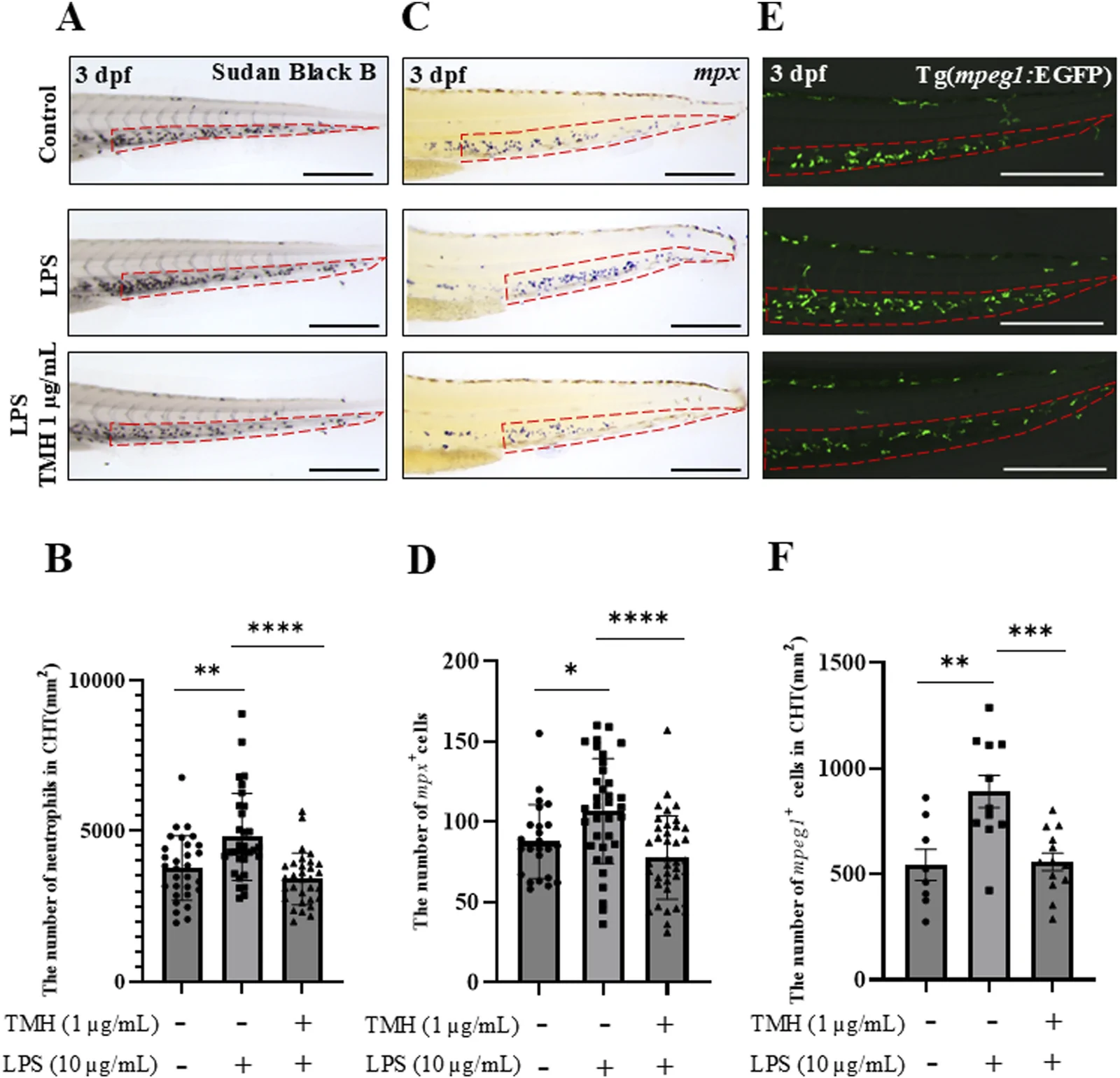
TMH suppresses LPS-induced inflammatory cell recruitment in zebrafish embryos. **(A)** Representative images of neutrophil recruitment in the CHT in 3 dpf zebrafish embryos following LPS stimulation, assessed by Sudan Black B staining. Top panel: control embryos treated with 0.0025% DMSO (n = 29); middle panel: LPS-treated embryos (10 μg/mL; n = 32); bottom panel: embryos co-treated with LPS (10 μg/mL) and TMH (1 μg/mL; n = 33). **(B)** Quantification of neutrophil accumulation in the CHT following LPS stimulation with or without TMH treatment using Sudan Black B staining from **(A)**. **(C)** Representative images of whole-mount *in situ* hybridization showing *mpx*-positive neutrophils in 3 dpf embryos under LPS and TMH treatment conditions. Top panel (DMSO 0.0025%; n = 34); middle panel (LPS 10 μg/mL; n = 35); bottom panel (LPS 10 μg/mL with TMH 1 μg/mL; n = 39). **(D)** Quantification of *mpx*-positive cells within the CHT from **(C)**. **(E)** Representative confocal images of macrophages in 3 dpf Tg (*mpeg1*:EGFP) zebrafish embryos following LPS and TMH treatment. Top panel (DMSO 0.0025%; n = 8); middle panel (LPS 10 μg/mL; n = 11); bottom panel (LPS 10 μg/mL with TMH 1 μg/mL; n = 13). **(F)** Quantification of *mpeg1*:EGFP-positive macrophages in the CHT region shown in **(E)**. Red dashed outline indicates the CHT. All data are presented as mean ± SEM. Statistical significance was determined using an unpaired two-tailed Student’s t-test. *P < 0.05; **P < 0.01; ***P < 0.05; ****P < 0.0001. Scale bars, 300 µm.

The anti-inflammatory effects of TMH were evaluated using an LPS-induced zebrafish model of inflammation (Yang et al., 2014; Lee et al., 2024b). LPS stimulation markedly increased neutrophil recruitment to the CHT, whereas co-treatment with 1 μg/mL TMH markedly reduced LPS-induced neutrophil accumulation, as assessed using Sudan Black B staining (Figures 5A,B).

These findings were further supported by whole-mount *in situ* hybridization using a neutrophil-specific *mpx* probe (Lieschke et al., 2001), which revealed a clear reduction in *mpx*-positive cells following TMH treatment (Figures 5C,D). A lower concentration of TMH (0.5 μg/mL) had a weaker inhibitory effect, suggesting a dose-dependent anti-inflammatory response (Supplementary Figure 2).

Macrophage recruitment was assessed using live imaging of Tg (*mpeg1*:EGFP) zebrafish larvae. LPS exposure increased the number of macrophages in the CHT, whereas TMH treatment attenuated this response (Figures 5E,F). These *in vivo* results demonstrate that TMH effectively suppresses inflammatory cell recruitment, consistent with its inhibitory effects on NLRP3 inflammasome activation in macrophages *in vitro*. Notably, the effective concentrations differed substantially between *in vitro* and *in vivo* experiments. While higher concentrations were required to observe direct cellular effects *in vitro*, lower doses were sufficient to produce anti-inflammatory outcomes in the zebrafish model, likely reflecting differences in bioavailability, metabolism, and systemic distribution.

### 3.6 UPLC-QTOF-MS profiling characterizes the phytochemical composition of TMH

**FIGURE 6:**
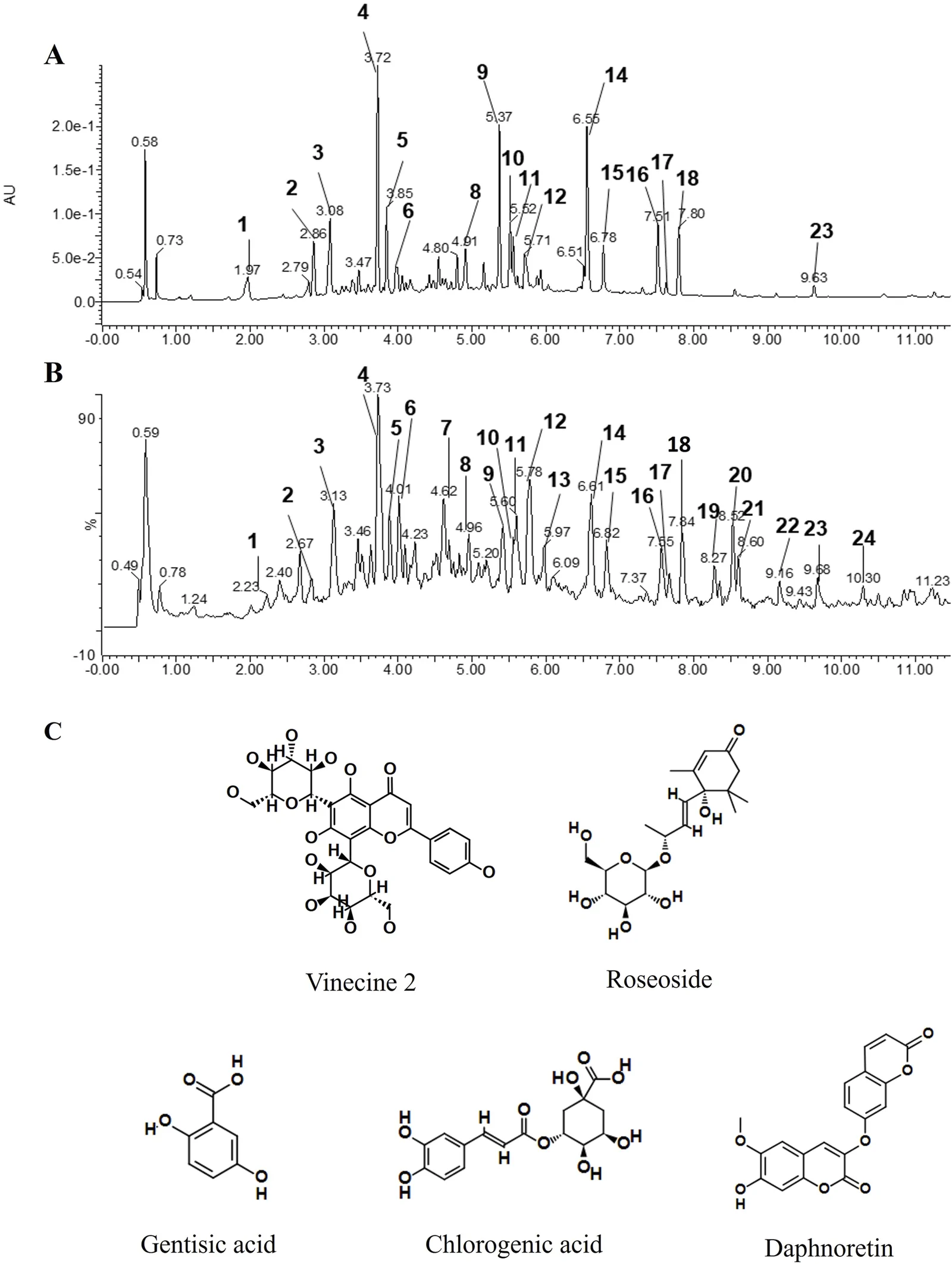
Representative chromatographic profiles of the TMH. **(A)** UV chromatogram monitored at 254 nm. **(B)** Total ion chromatogram (TIC) obtained by UPLC–QTOF–MS analysis. **(C)** Representative chemical structures of selected metabolites tentatively identified from the TMH by UPLC–QTOF–MS analysis.

UPLC–QTOF–MS analysis detected multiple metabolite features in TMH, among which 24 metabolites were tentatively identified based on accurate mass measurements and MS/MS fragmentation patterns (Figures 6A,B). The chemical structures of the three metabolites exhibiting the highest MS signal intensities are presented in Figure 6C.

Based on the relative peak intensities observed in the MS chromatograms, flavonoids were identified as the predominant class of metabolites in the extract. Among them, metabolite 4 (Vicenin 2), 9 (Acacetin-hexoside-pentoside), and 14/15 (Kaempferol-O-rutinoside isomers) exhibited the highest signal intensities, indicating that glycosylated flavones and flavonols constitute the major phytochemical metabolites. metabolite 16 (Chrysoeriol), a flavone aglycone, was also detected at a relatively high level.

Phenolic acids and related derivatives, including metabolites 1 (Gentisic acid), 2 (p-Coumaric acid 4-[apiosyl-(1→2)-glucoside]), and 3 (Chlorogenic acid), were present at intermediate signal levels. Additional phenolic metabolites such as metabolites 17 (Triumbelletin) and 18 (Daphnoretin) further support the predominance of polyphenolic scaffolds in TMH. The monoterpenoid glycoside metabolites 5 (Roseoside) was detected at moderate relative intensity. Detailed mass information supporting the tentative identification is summarized in Supplementary Table S1.

## 4 Discussion

In this study, we demonstrated that TMH suppresses NLRP3 inflammasome activation in macrophages and exerts anti-inflammatory effects *in vivo*. Our data suggest that TMH interferes with inflammasome assembly, as reflected by reduced ASC oligomerization and decreased IL-1β secretion. Notably, this suppression was associated with the attenuation of NLRP3 ATPase activity, a prerequisite for the conformational changes and oligomerization of NLRP3. Because ATP binding and hydrolysis within the NACHT domain are essential for inflammasome assembly, modulation of this enzymatic activity may influence downstream ASC oligomerization. While synthetic inhibitors like MCC950 directly target the NLRP3 ATPase function (Tapia-Abellán et al., 2019; Li et al., 2022), TMH is a complex botanical extract; thus, the observed reduction in ATPase activity may reflect indirect or allosteric modulation. Future studies employing target engagement assays, such as molecular docking, DARTS, or CETSA, will be necessary to determine whether specific metabolites may interact with conserved Walker A/B motifs or influence NACHT conformational dynamics.

A common limitation in botanical research is attributing pharmacological activity to broad classes of secondary metabolites (e.g., general flavonoids or phenolic acids), which are ubiquitous in many medicinal plants. To address this concern, our UPLC–QTOF–MS profiling enabled the identification of the most abundant individual metabolites in TMH, including vicenin-2, kaempferol-O-rutinoside, chrysoeriol, acacetin derivatives, and chlorogenic acid. Some of the identified metabolites have previously been reported in TMH (Zhong et al., 2019; Feki et al., 2025), whereas others have not yet been described from this species. This metabolites-level resolution provides a more defined phytochemical basis for the observed bioactivity. Notably, several of these metabolites have been reported to modulate inflammasome-associated signaling pathways. For example, chlorogenic acid (Bao et al., 2025) and kaempferol derivatives (Abdulaal et al., 2024; Wang et al., 2025) have been shown to attenuate NLRP3-dependent IL-1β secretion in macrophages, while flavones such as chrysoeriol and acacetin analogs exhibit anti-inflammatory effects in cellular models (Wang and Hong, 2022; Bu et al., 2024). Furthermore, non-flavonoid metabolites such as the monoterpenoid glycoside identified in TMH (metabolite 5, roseoside) may also contribute to the observed inflammasome modulation. Previous studies have shown that certain monoterpenoid glycosides, such as paeoniflorin, attenuate NLRP3 inflammasome activation and IL-1β secretion in inflammatory models (Liu et al., 2020; Zhang et al., 2026). Therefore, it is plausible that the monoterpenoid glycoside present in TMH may modulate NLRP3-associated signaling.

Although the present study does not establish direct target engagement between these metabolites and NLRP3, their structural features and documented bioactivities suggest that they may contribute to the observed suppression of inflammasome assembly. Rather than reflecting a nonspecific antioxidant effect, the enriched presence of glycosylated flavones and flavonols in TMH may underlie its relative selectivity toward NLRP3-associated pathways. It is therefore plausible that the combined and potentially synergistic actions of these predominant metabolites account for the overall inhibitory profile observed in this study.

*In vivo*, TMH attenuated neutrophil and macrophage recruitment in a zebrafish model of acute inflammation at concentrations without developmental toxicity. However, zebrafish models primarily reflect acute innate immune responses and may not fully recapitulate mammalian chronic inflammatory conditions (Novoa and Figueras, 2011). Another limitation is the difference between the effective concentrations observed *in vitro* and the doses used in the zebrafish model. The IC_50_ for inhibition of IL-1β secretion *in vitro* was approximately 9.4 μg/mL, whereas the maximum dose used *in vivo* was limited to 1 μg/mL due to toxicity. This discrepancy highlights the need for future studies to identify the active metabolites and optimize dosing strategies. Furthermore, a direct causal link between NLRP3 inhibition and the *in vivo* phenotype requires future confirmation by assessing NLRP3 activation and IL-1β expression at the protein level in zebrafish (Li et al., 2020).

Importantly, TMH did not substantially alter intracellular ROS production or NF-κB signaling, supporting a more targeted effect on NLRP3-associated pathways rather than broad upstream immunosuppression. In conclusion, TMH represents a botanical extract capable of modulating NLRP3 inflammasome activation. Future bioassay-guided fractionation to isolate active principles, combined with validation in mammalian disease models, will be essential to advance *T. hirsuta*–derived candidates toward therapeutic development for NLRP3-driven inflammatory disorders.

## Data Availability

The original contributions presented in the study are included in the article/Supplementary Material, further inquiries can be directed to the corresponding authors.

## Supplementary material

SUPPLEMENTARY FIGURE S1 TMH has no effect on pro-IL-1β expression. **(A)** LPS-primed J774A.1 cells were treated with TMH for 2 h and stimulated with ATP (5 mM) for 30 min, with or without MCC950. Soluble lysates were analyzed by immunoblotting for pro-IL-1β and α-tubulin. **(B,C)** PMA-differentiated THP-1 cells were primed with LPS, treated with TMH for 2 h, and stimulated with ATP (5 mM) **(B)** or nigericin (10 μM) **(C)** for 1 h, in the presence or absence of MCC950. Soluble lysates were subjected to immunoblot analysis.

SUPPLEMENTARY FIGURE S2 TMH does not alter neutrophil counts at concentrations below 1 μg/mL. **(A)** Representative images from whole-mount *in situ* hybridization show that LPS-induced neutrophils are reduced at 0.5 μg/mL and 1 μg/mL after TMH treatment at 3 dpf. Control (n = 16), LPS (n = 15), TMH 0.5 μg/mL (n = 20), TMH 1 μg/mL (n = 17) including LPS (10 μg/mL) respectively. **(B)** *mpx* -positive cells numbers in the CHT region of 3 dpf embryos are quantified. **(C)** Representative images showing the recruitment of neutrophils in CHT after TMH treatment at 3 dpf by LPS and Sudan Black B staining. The upper panel is the control (n = 14), the second panel is LPS (10 μg/mL) containing 0.0025% DMSO (n = 15). The third and last panels were treated simultaneously with LPS (10 μg/mL) and TMH 0.5 μg/mL (n = 19) and TMH 1 μg/mL (n = 18), respectively. **(D)** Quantify by Sudan Black B staining that the LPS-induced inflammatory response is reduced after TMH treatment. All graphs display the mean ± S.E.M. of individual data points. P-values were determined using an unpaired two-tailed Student’s t-test. *P& **P& ***P < 0.05; ****P < 0.0001. All scale bars represent 300 µm.
