# Correction: *Thymelaea hirsuta* (L.) Endl. extract attenuates NLRP3 inflammasome activation via modulation of ATPase activity

**DOI:** 10.3389/fphar.2026.2010669

**Published:** 2026-10-09

**Authors:** Seongjong Lee, Seoyeon Jang, Hangyeol Lee, Ali Khorchani, Abdelhamid Khaldi, Mi Jin Lee, Dong Keun Yi, Soo-Yong Kim, Sangho Choi, Hyungseok Seo, Man S. Kim, Jin Hyub Paik, Hyung Won Ryu, Yoonsung Lee, Yong Hwan Park

**Affiliations:** 1 BK21 R&E Initiative for Advanced Precision Medicine, Ajou University School of Medicine, Suwon, Republic of Korea; 2 Department of Microbiology, Ajou University School of Medicine, Suwon, Republic of Korea; 3 Department of Biomedical Sciences, Graduate School of Ajou University, Suwon, Republic of Korea; 4 Department of Biomedical Science and Technology, Kyung Hee University, Seoul, Republic of Korea; 5 School of Medicine, Clinical Research Institute, Kyung Hee University Hospital at Gangdong, Kyung Hee University, Seoul, Republic of Korea; 6 Natural Product Research Center and Natural Product Central Bank, KRIBB, Cheongju-si, Chungcheongbuk-do, Republic of Korea; 7 Laboratory of Management and Valorization of Forest Resources, The National Research Institute of Rural Engineering, Water, and Forestry. INRGREF. University of Carthage, Ariana, Tunisia; 8 International Biological Material Research Center, Korea Research Institute of Bioscience and Biotechnology, Daejeon, Republic of Korea; 9 Department of Anesthesiology and Pain Medicine, Kyung Hee University Hospital at Gangdong, College of Medicine, Kyung Hee University, Seoul, Republic of Korea; 10 Center for Space Biomedical Sciences, NEXUS Institute, Kyung Hee University, Seoul, Republic of Korea

**Keywords:** apoptosis-associated speck-like protein containing a CARD, caspase-1, interleukin-1β, NLRP3 inflammasome, *Thymelaea hirsuta* (L.) Endl

An incorrect Funding statement was provided. The correct funding statement reads:

The author(s) declared that financial support was received for this work and/or its publication. This work was supported by the National Research Foundation of Korea (NRF) grants funded by the Korean government (MSIT) (RS-2025-24534393 and RS-2023-00217595 to Y.H.P.) and a grant of the Korea Health Technology R&D Project through the Korea Health Industry Development Institute (KHIDI), funded by the Ministry of Health & Welfare, Republic of Korea (RS-2022-KH130309 and RS-2024-00407544 to Y.H.P). This work was also supported by the National Research Foundation of Korea (NRF) grants funded by the Korean government (MSIT) (RS-2022-NR069341 and RS-2022-NR070587 to Y.L.) and Global - Learning & Academic research institution for Master’s·PhD students, and Postdocs (G-LAMP) Program of the NRF grant funded by the Ministry of Education (No. RS-2025-25442355 to Y.L.).

This work was also supported by the collaborative research agreement between the National Institute for Research in Rural Engineering, Water and Forests (INRGREF) and the Korea Research Institute of Bioscience and Biotechnology (KRIBB). The authors acknowledge KRIBB for financial support and INRGREF for facilitating the implementation of the study.

The original version of this article has been updated.

