# Supplementary material for: Thymelaea hirsuta (L.) Endl. extract attenuates NLRP3 inflammasome activation via modulation of ATPase activity
Source: Front Pharmacol. 2026 Apr 15;17:1781860. doi: 10.3389/fphar.2026.1781860 (PMC13125143; doi:10.3389/fphar.2026.1781860)
Supplement: Supplementary file 1 [file Table1.DOCX]

**Table S1.** Tentative characterization of constituents in *Thymelaea hirsuta* (L.) Endl. extract using UPLC–QTOF–MS.

| **No.** | **RT** | **Tentative ID** | **Formula** | **Calculated *m/z*** | **Detected *m/z*** | **error** | **Adduct** | **Ref.** |
| --- | --- | --- | --- | --- | --- | --- | --- | --- |
| 1 | 2.01 | Gentisic acid | C7H6O4 | 153.0188 | 153.0183 | -3.16 | -H | (Wang et al., 2013) |
| 2 | 2.83 | p-Coumaric acid 4-[apiosyl-(1->2)-glucoside] | C20H26O12 | 458.14243 | 457.134 | -1.32 | -H | (Bagatini et al., 2023) |
| 3 | 3.13 | Chlorogenic acid | C16H18O9 | 353.08726 | 353.0874 | 0.4 | -H | (Feki et al., 2025) |
| 4 | 3.75 | Vicenin 2 | C27H30O15 | 594.15847 | 593.1507 | 0.1 | -H | (Feki et al., 2025) |
| 5 | 3.88 | Roseoside | C19H30O8 | 385.18624 | 385.1868 | 1.45 | -H | (Feki et al., 2025) |
| 6 | 4.01 | unknown sulfohexoside | C19H30O11S | 465.1431 | 465.1425 | -1.2 | -H | (Hsu and Turk, 2004) |
| 7 | 4.62 | Phenethyl-sulfohexoside | C14H20O9S | 363.075 | 363.0757 | 1.99 | -H | (Gage et al., 1992) |
| 8 | 4.96 | edgeworn or daphnogitin | C18H10O6 | 321.0399 | 321.0403 | 1.21 | -H | (Wu et al., 2022) |
| 9 | 5.42 | Acacetin-hexoside-pentoside | C27H30O14 | 577.15573 | 577.1568 | 1.85 | -H | (Feki et al., 2025) |
| 10 | 5.56 | Chrysoeriol 7-O-β-D-glucoside | C22H22O11 | 461.1084 | 461.1086 | 0.46 | -H | (Castro et al., 2026) |
| 11 | 5.6 | 3-oxo-α-ionol O-glucoside sulfate (isomer) | C19H30O10S | 449.14477 | 449.1487 | 8.75 | -H | (Shi et al., 2022) |
| 12 | 5.78 | 3-oxo-α-ionol O-glucoside sulfate (isomer) | C19H30O10S | 449.14477 | 449.148 | 7.19 | -H | (Shi et al., 2022) |
| 13 | 5.97 | Oleuropein | C25H32O13 | 539.1765 | 539.1779 | 2.66 | -H | (Li et al., 2025) |
| 14 | 6.61 | Kaempferol-O-rutinoside (isomer) | C30H26O13 | 593.12952 | 593.1299 | 0.65 | -H | (Feki et al., 2025) |
| 15 | 6.82 | Kaempferol-O-rutinoside (isomer) | C30H26O13 | 593.12952 | 593.1308 | 2.17 | -H | (Feki et al., 2025) |
| 16 | 7.55 | Chrysoeriol | C16H12O6 | 299.0556 | 299.0553 | -0.88 | -H | (Zhong et al., 2019) |
| 17 | 7.67 | Triumbelletin | C27H14O9 | 481.056 | 481.0556 | -0.74 | -H | (Wang et al., 2024) |
| 18 | 7.84. | Daphnoretin | C19H12O7 | 351.0505 | 351.0506 | 0.35 | -H | (Mi et al., 2023) |
| 19 | 8.27 | 9,12,13-TriHOME | C18H34O5 | 329.2328 | 329.233 | 0.61 | -H | (Crescenzi et al., 2025) |
| 20 | 8.52 | Dihydroxyhexadecanoic acid | C16H32O4 | 287.2222 | 287.2226 | 1.27 | -H | (Otify et al., 2015) |
| 21 | 8.6 | Putative 3′,3′′′-binaringenin (isomer) | C30H22O10 | 541.1135 | 541.1136 | 0.24 | -H | (Yao et al., 2017) |
| 22 | 9.16 | Putative 3′,3′′′-binaringenin (isomers) | C30H22O10 | 541.1135 | 541.1138 | 0.61 | -H | (Yao et al., 2017) |
| 23 | 9.68 | Methylapigenin | C16H12O5 | 283.0606 | 283.0606 | -0.17 | -H | (Okińczyc et al., 2021) |
| 24 | 10.28 | 12,13 -DiHODE | C18H32O4 | 311.2222 | 311.2224 | 0.53 | -H | (Ostermann et al., 2015) |

**References**

Castro, J.W.G., Souza, G.G.d.O., da Costa, J.G.M., and Rodrigues, F.F.G. (2026). Citrus reticulata Blanco: A Review on Chemical Composition and Biological Activities. *Chemistry & Biodiversity* 23(1)**,** e03156. doi: 10.1002/cbdv.202503156.

Crescenzi, M.A., Gallart-Ayala, H., Stellato, C., Popolo, A., Ivanisevic, J., Piacente, S., et al. (2025). A targeted mass spectrometric approach to evaluate the anti-inflammatory activity of the major metabolites of Foeniculum vulgare Mill. waste in human bronchial epithelium. *Molecules* 30(7)**,** 1407. doi: 10.3390/molecules30071407.

Feki, I., Hadrich, F., Mahmoudi, A., Llorent-Martínez, E.J., Bouallagui, Z., Souilem, S., et al. (2025). Thymelaea hirsuta extract attenuates testosterone-induced benign prostatic hyperplasia in rats: Effect on oxidative stress, inflammation and apoptosis. *Journal of Ethnopharmacology* 342**,** 119373. doi: 10.1016/j.jep.2025.119373.

Gage, D.A., Huang, Z.-H., and Benning, C. (1992). Comparison of sulfoquinovosyl diacylglycerol from spinach and the purple bacterium Rhodobacter sphaeroides by fast atom bombardment tandem mass spectrometry. *Lipids* 27(8)**,** 632-636. doi: 10.1007/BF02536123.

Hsu, F.-F., and Turk, J. (2004). Studies on sulfatides by quadrupole ion-trap mass spectrometry with electrospray ionization: structural characterization and the fragmentation processes that include an unusual internal galactose residue loss and the classical charge-remote fragmentation. *Journal of the American Society for Mass Spectrometry* 15(4)**,** 536-546. doi: 10.1016/j.jasms.2003.12.007.

Li, H., Deng, N., Yang, J., Zhao, Y., Jin, X., Cai, A., et al. (2025). Anti-inflammatory and antioxidant properties of oleuropein in human keratinocytes characterized by bottom-up proteomics. *Frontiers in Pharmacology* 15**,** 1496078. doi: 10.3389/fphar.2024.1496078.

Mi, H., Zhang, P., Yao, L., Gao, H., Wei, F., Lu, T., et al. (2023). Identification of Daphne Genkwa and its vinegar-processed products by ultraperformance liquid chromatography–quadrupole time-of-flight mass spectrometry and chemometrics. *Molecules* 28(10)**,** 3990. doi: 10.3390/molecules28103990.

Okińczyc, P., Widelski, J., Szperlik, J., Żuk, M., Mroczek, T., Skalicka-Woźniak, K., et al. (2021). Impact of plant origin on eurasian propolis on phenolic profile and classical antioxidant activity. *Biomolecules* 11(1)**,** 68. doi: 10.3390/biom11010068

Full text linksCite.

Ostermann, A.I., Willenberg, I., and Schebb, N.H. (2015). Comparison of sample preparation methods for the quantitative analysis of eicosanoids and other oxylipins in plasma by means of LC-MS/MS. *Analytical and bioanalytical chemistry* 407(5)**,** 1403-1414. doi: 10.1007/s00216-014-8377-4.

Otify, A., George, C., Elsayed, A., and Farag, M.A. (2015). Mechanistic evidence of Passiflora edulis (Passifloraceae) anxiolytic activity in relation to its metabolite fingerprint as revealed via LC-MS and chemometrics. *Food & Function* 6(12)**,** 3807-3817. doi: 10.1039/c5fo00875a.

Shi, L., Zhao, W., Yang, Z., Subbiah, V., and Suleria, H.A.R. (2022). Extraction and characterization of phenolic compounds and their potential antioxidant activities. *Environmental Science and Pollution Research* 29(54)**,** 81112-81129. doi: <https://doi.org/10.1007/s11356-022-23337-6>.

Wang, E., Zhou, Y., Miao, X., He, G., Lv, P., Wang, L., et al. (2024). Facilitating effects of plant extracts on soil health and replanted Panax ginseng growth in recession soil. *Plos one* 19(10)**,** e0311679. doi: 10.1371/journal.pone.0311679.

Wang, L., Halquist, M.S., and Sweet, D.H. (2013). Simultaneous determination of gallic acid and gentisic acid in organic anion transporter expressing cells by liquid chromatography–tandem mass spectrometry. *Journal of Chromatography B* 937**,** 91-96. doi: 10.1016/j.jchromb.2013.08.024.

Wu, M., Su, X., Wu, Y., Luo, Y., Guo, Y., and Xue, Y. (2022). Glycosylated coumarins, flavonoids, lignans and phenylpropanoids from Wikstroemia nutans and their biological activities. *Beilstein Journal of Organic Chemistry* 18(1)**,** 200-207. doi: 10.3762/bjoc.18.23.

Yao, H., Chen, B., Zhang, Y., Ou, H., Li, Y., Li, S., et al. (2017). Analysis of the total biflavonoids extract from Selaginella doederleinii by HPLC-QTOF-MS and its in vitro and in vivo anticancer effects. *Molecules* 22(2)**,** 325. doi: 10.3390/molecules22020325.

Zhong, J.-L., Muhammad, N., Gu, Y.-C., and Yan, W.-D. (2019). A simple and efficient method for enrichment of cocoa polyphenols from cocoa bean husks with macroporous resins following a scale-up separation. *Journal of food engineering* 243**,** 82-88. doi: <https://doi.org/10.1016/j.jfoodeng.2018.08.023>.
