# Supplementary figures and images for: Thymelaea hirsuta (L.) Endl. extract attenuates NLRP3 inflammasome activation via modulation of ATPase activity
Source: Front Pharmacol. 2026 Apr 15;17:1781860. doi: 10.3389/fphar.2026.1781860 (PMC13125143; doi:10.3389/fphar.2026.1781860)

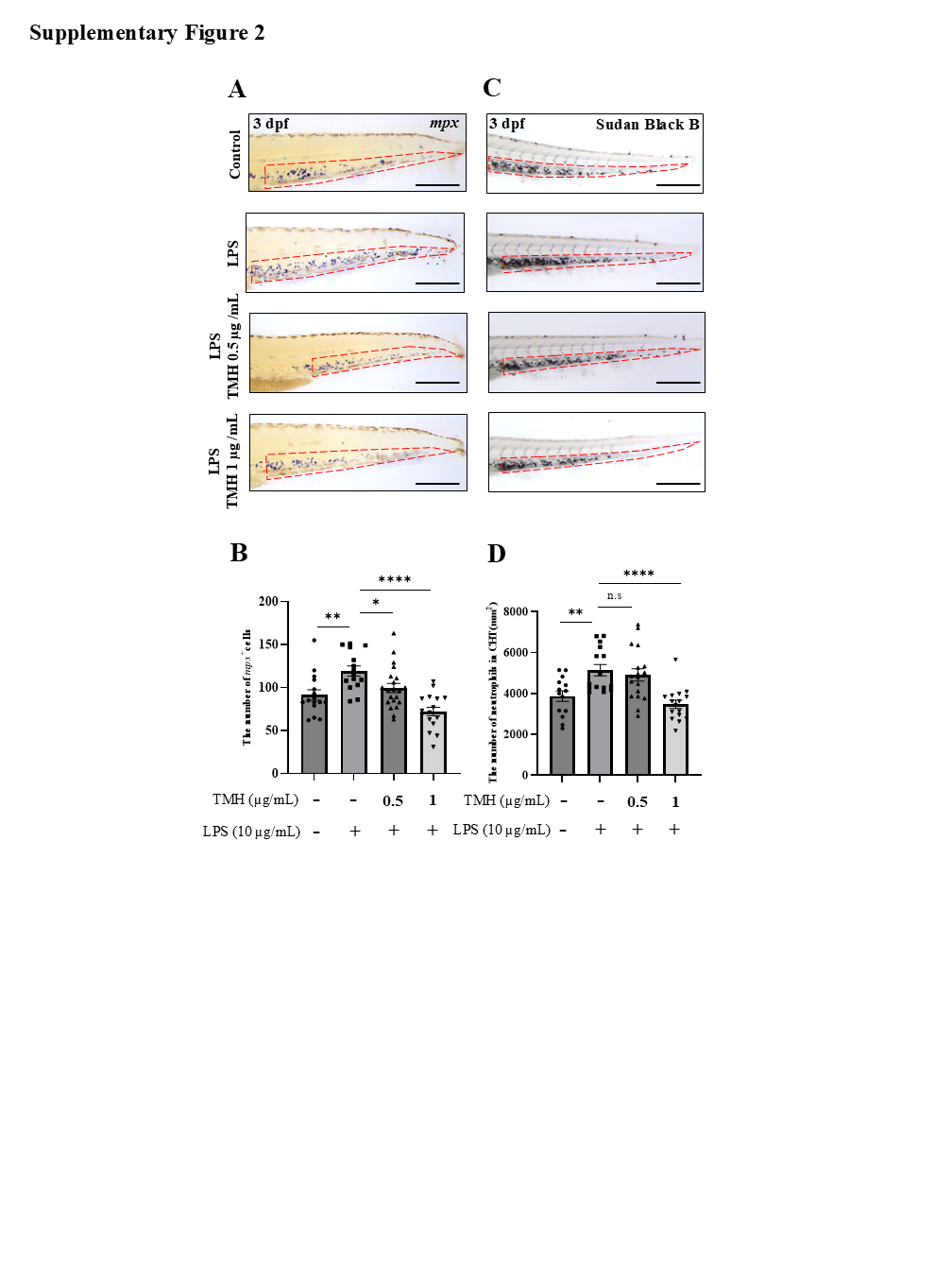

Supplement: Supplementary file 2 [file Image2.TIF]

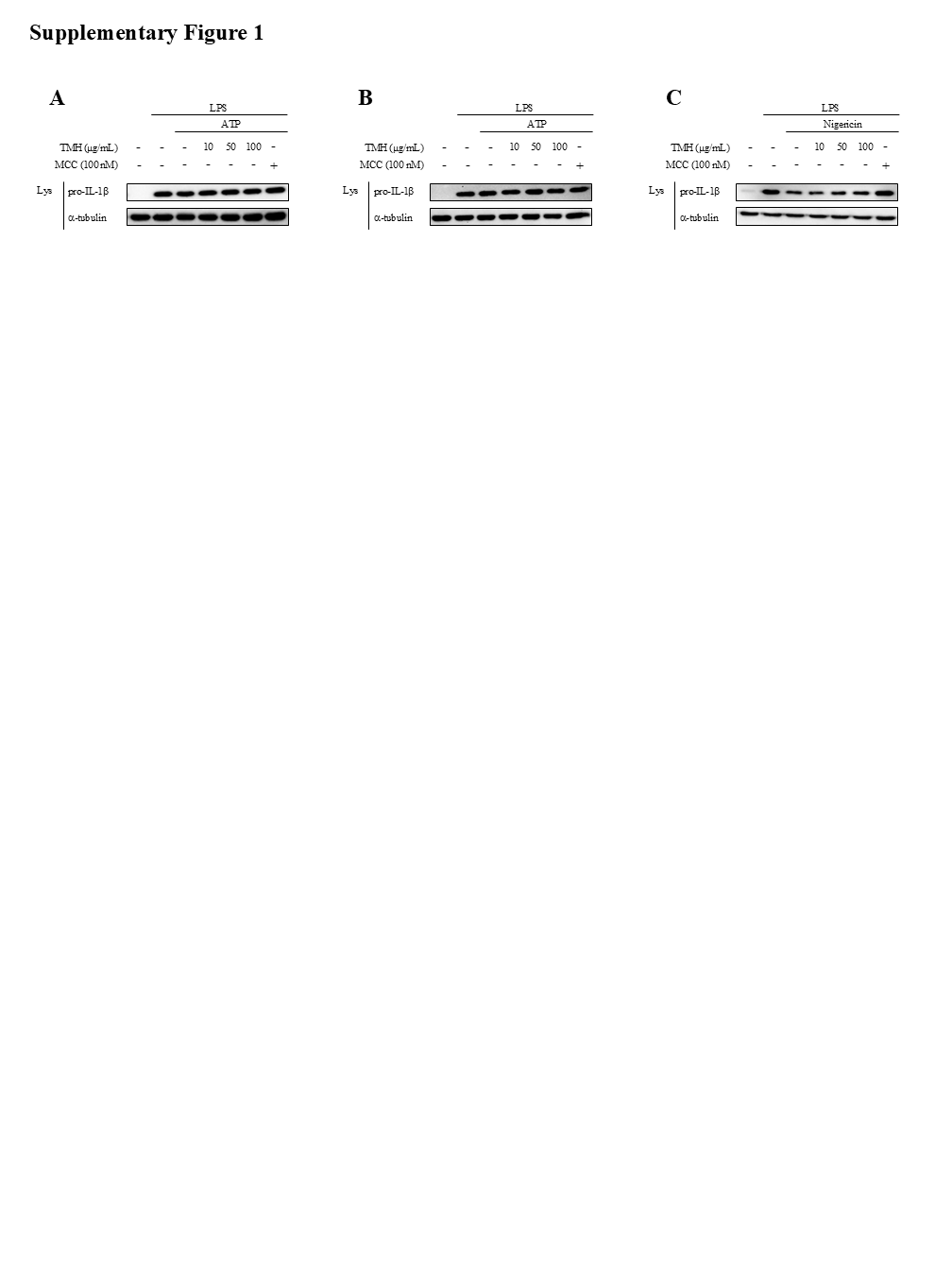

Supplement: Supplementary file 3 [file Image1.tif]
